# A Complex, Convexity, Anterior Superior Sagittal Sinus Dural Arteriovenous Fistula Secondary to Venous Sinus Thrombosis

**DOI:** 10.7759/cureus.76241

**Published:** 2024-12-23

**Authors:** Rajan Chand, Rabeeia Parwez, Nathan Chan, Lakshmi Kanagrajah, Raghu Vindlacheruvu, Lauren Harris

**Affiliations:** 1 Neurosurgery, Barts and The London School of Medicine and Dentistry, Queen Mary University of London (QMUL), London, GBR; 2 Neurosurgery, Queens Hospital Center, Romford, GBR; 3 Interventional Neuroradiology, University College London, London, GBR; 4 Interventional Neuroradiology, Queens Hospital Center, Romford, GBR

**Keywords:** coeliac disease, craniotomy, dural arteriovenous fistula, interventional radiology, superior sagittal sinus, venous sinus thrombosis

## Abstract

We report the management of a convexity dural arteriovenous fistula (dAVF) in an uncommon anterior superior sagittal sinus (SSS) location. This was a high-risk Cognard IIa+b dAVF, which is notoriously complex to treat. Endoscopic management alone for complex SSS dAVFs is challenging due to the often bilateral arterial supply to the fistula, as demonstrated in this case. Hence, proceeding straight to surgery is a viable option, skipping the need for interventional radiological (IR) coiling. This is the first reported case of a fistula secondary to venous sinus thrombosis in a coeliac patient. Our patient initially underwent endoscopic treatment, which failed due to arterial supply from other arteries continuing to supply the fistula. The patient then underwent a midline craniotomy where the feeding arteries were ligated, followed by division of the anterior third of the SSS, allowing further division and ligation of arterial feeders. It is essential to be aware of the prothrombotic state of coeliacs and the risk of thrombosis and fistula formation. This allows physicians to recognize the signs of raised intracranial pressure, facilitating early intervention.

## Introduction

Dural arteriovenous fistulas (dAVFs) are brain or spinal cord vascular abnormalities where an artery connects directly to a dural vein or venous sinus [1]. They may be idiopathic or secondary to trauma, iatrogenic, inflammation, or venous sinus thrombosis [2]. They differ from arteriovenous malformations (AVMs), being durally based, with no parenchymal nidus [3]. They account for 10-15% of all cerebral vascular malformations within the brain, often presenting at 40-60 years of age [1]. They are most commonly situated at the transverse-sigmoid junction and present with headache and pulsatile tinnitus [1,4]. Vascular imaging is required to characterize the fistula, and a six-vessel digital subtraction angiogram (DSA) is the gold standard [4]. 

dAVFs are classified by the Borden or Cognard classification systems into low and high-risk groups. Type I Borden, or type I and IIa Cognard, has venous drainage directly into a dural sinus and is low risk, often managed conservatively. Higher grades, with cortical venous drainage, are more aggressive. Risks include hemorrhage and venous hypertension, with its associated sequelae such as raised intracranial pressure, which is characterized by symptoms such as complex headaches, papilloedema, and tinnitus [1,5]. If appropriately fit, these patients are candidates for intervention, with treatment options including endovascular embolization, open surgery, or rarely stereotactic radiosurgery (SRS) [1]. This should be determined by a dedicated neurovascular multidisciplinary team (MDT) meeting. Endovascular embolization is often the first line using liquid embolic agents or coils. This can be via the transarterial or transvenous route, occlusion at the site(s) of fistulation, to prevent recurrence and further arterial recruitment [1]. Surgery is often used if endovascular efforts are unlikely to be successful, for example, if access is difficult, or as a direct second line to divide the fistula. SRS is reserved for small, low-grade lesions in an attempt to encourage vessel thrombosis over months to years [6].

We present a case of a 67-year-old woman who presented with a complex Cognard IIa+b dAVF, likely secondary to extensive venous sinus thrombosis, on a background of coeliac disease. This is the first such case reported in the literature.

## Case presentation

A 67-year-old woman was referred to the neurosurgical department at Queen’s Hospital in 2021 following a magnetic resonance imaging (MRI) arranged by her primary care physician for investigation of global daily headache, neck stiffness, and left ear tinnitus of six months duration. Her headache was localized in the occipital region and would last all day and worsen at night. There were no aggravating or relieving factors; however, she would regularly take aspirin to try and relieve the headache. Accompanying symptoms were a feeling of heaviness in her head and losing her appetite, which resulted in her losing two stones in body weight. She would also complain of losing her balance; however, she did not have any falls.

Her past medical history included coeliac disease, epilepsy, osteoarthritis, bell's palsy, hypothyroidism, and two previous transient ischemic attacks (TIAs). Medication history included clopidogrel, thyroxine, atorvastatin, betahistine, and clonazepam. On examination, she was neurologically intact. 

**Figure 1:**
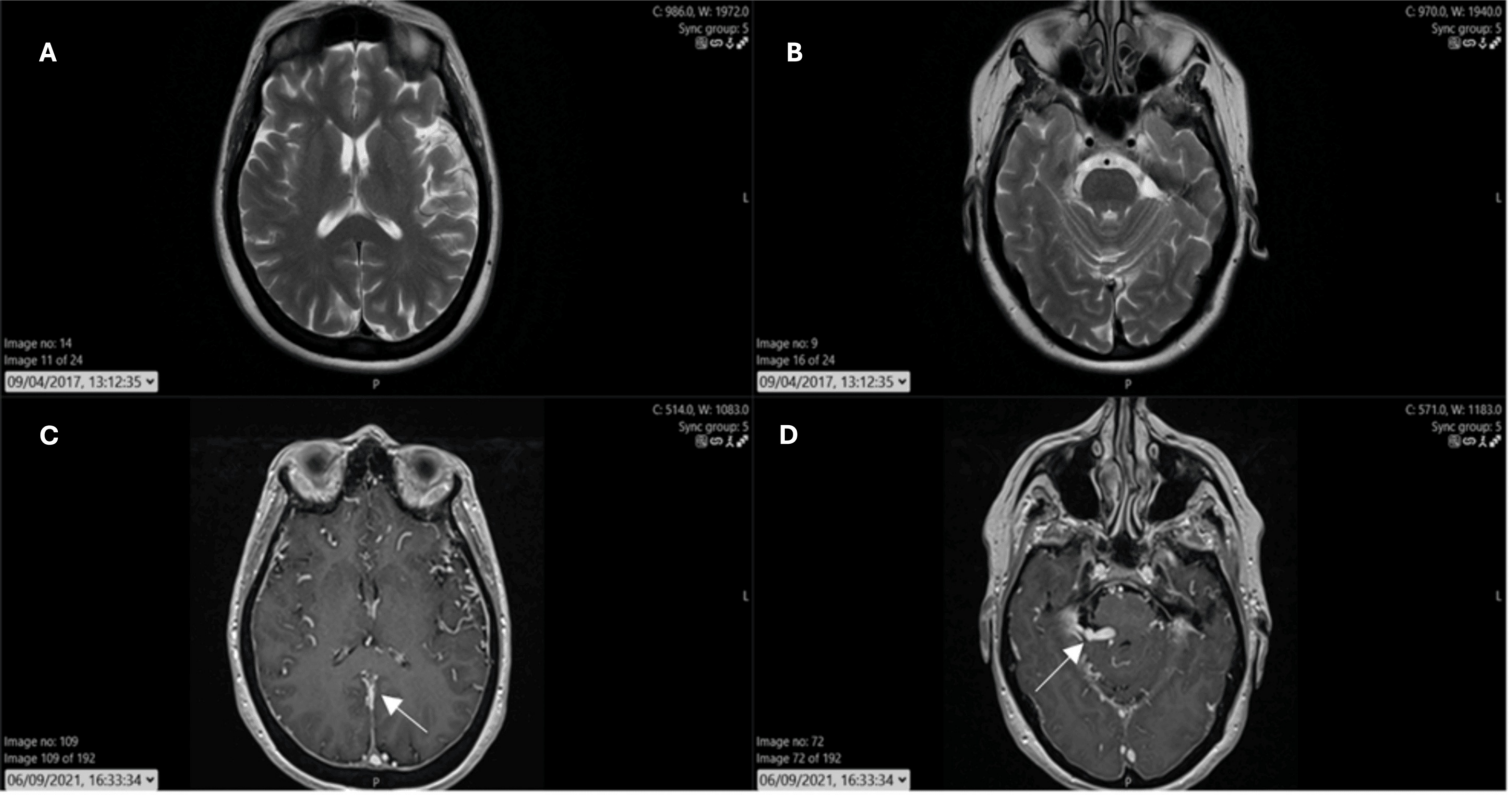
MRI brain. (A) and (B): T2 scan 2017 is grossly normal with no evidence of intracranial hypertension or vascular abnormality (unfortunately, no contrasted T1 available). (C) and (D): T1 with contrast scan 2021, demonstrating global venous engorgement, with dilated vessels supratentorially and infratentorially.

The MRI showed evidence of venous hypertension (Figure 1) and was grossly different from a previous MRI in 2017 (when investigated for her TIAs).

She was discussed at the neurovascular MDT meeting with a likely diagnosis of a dAVF. An urgent DSA for further evaluation was arranged. Clinically, she had progressive short-term memory loss whilst awaiting investigation. 

**Figure 2:**
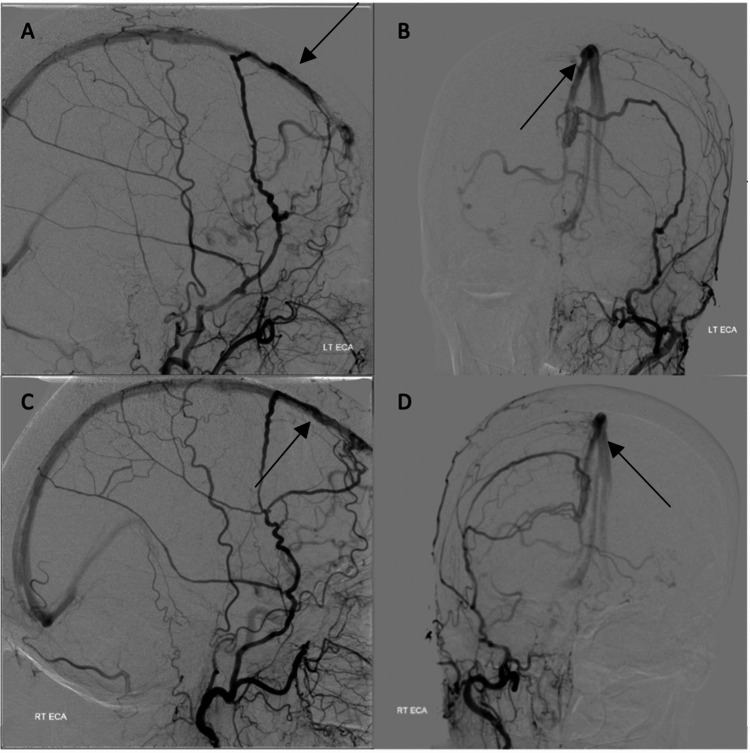
Digital subtraction angiogram. (A) and (B) Lateral and AP left ECA injection. (C) and (D) Lateral and AP right ECA injection. Fistula appreciated from MMA at anterior SSS with deep venous reflux. LT ECA: left external carotid artery, RT ECA: right external carotid artery, AP: anterior posterior, MMA: middle meningeal artery.

The DSA demonstrated a complex, convexity, anterior superior sagittal sinus (SSS) high-grade fistula. The predominant arterial supply was from the right external carotid artery (ECA) via the middle meningeal artery (MMA). There was a lesser supply from the left internal carotid artery (ICA), left ECA, and less significant right ICA pial supply. There was also a supply from the anterior falcine/ethmoidal arteries and the ophthalmic. The fistula drained into the rostral third of the SSS, with deep venous reflux into the right internal cerebral vein (Figure 2).

**Figure 3:**
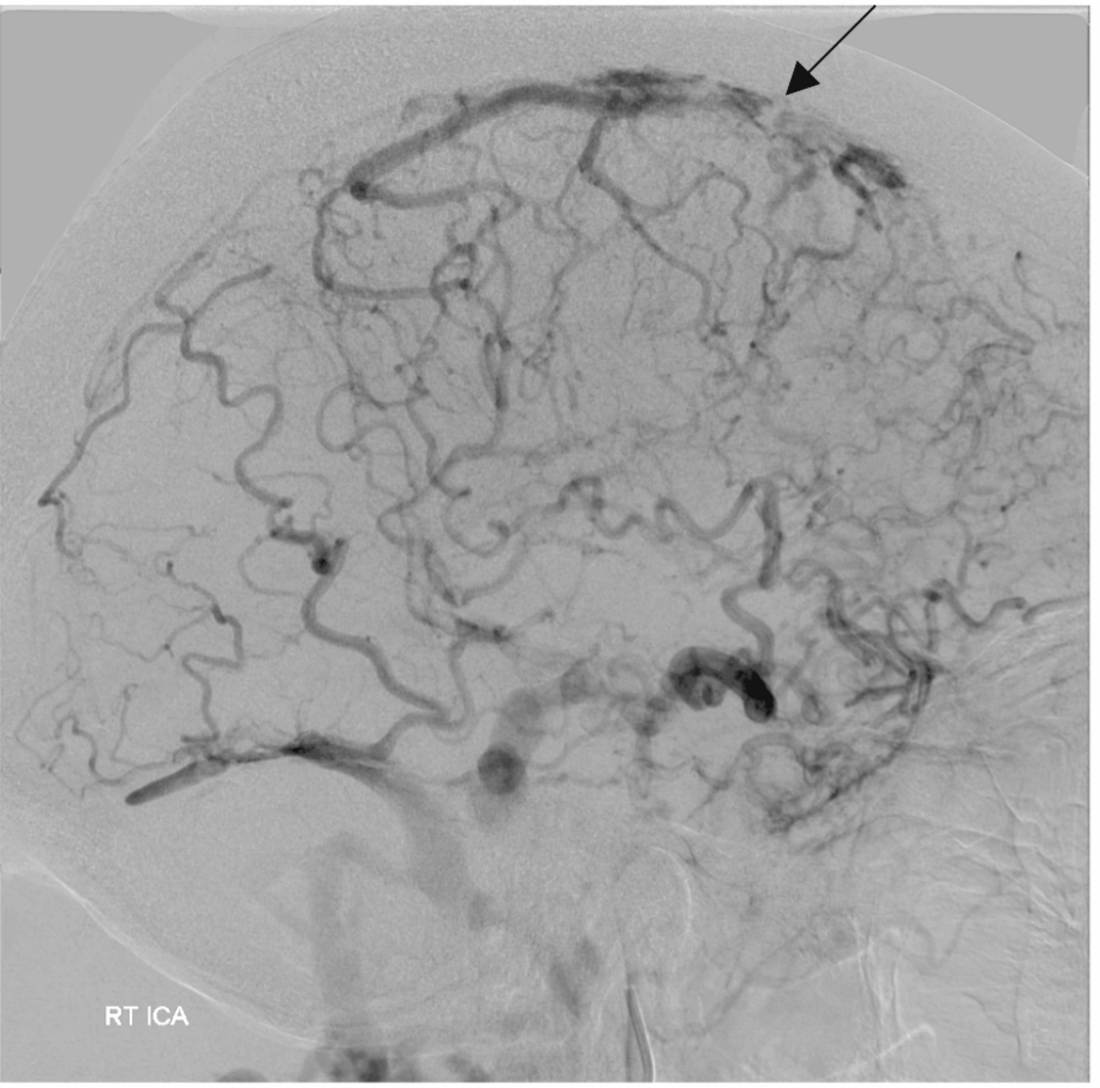
Digital subtraction angiogram. Venous phase demonstrating gross venous hypertension evident. RT ICA: right internal carotid artery.

She had gross venous hypertension (Figure 3). Her SSS was patent but had very high pressure due to the presence of the fistula. She had bilateral occlusion of both transverse-sigmoid sinuses. This meant she relied on the cavernous, cortical, pterygoid, facial, and peri mesencephalic venous drainage systems for venous outflow, as identified on MRI.

**Figure 4:**
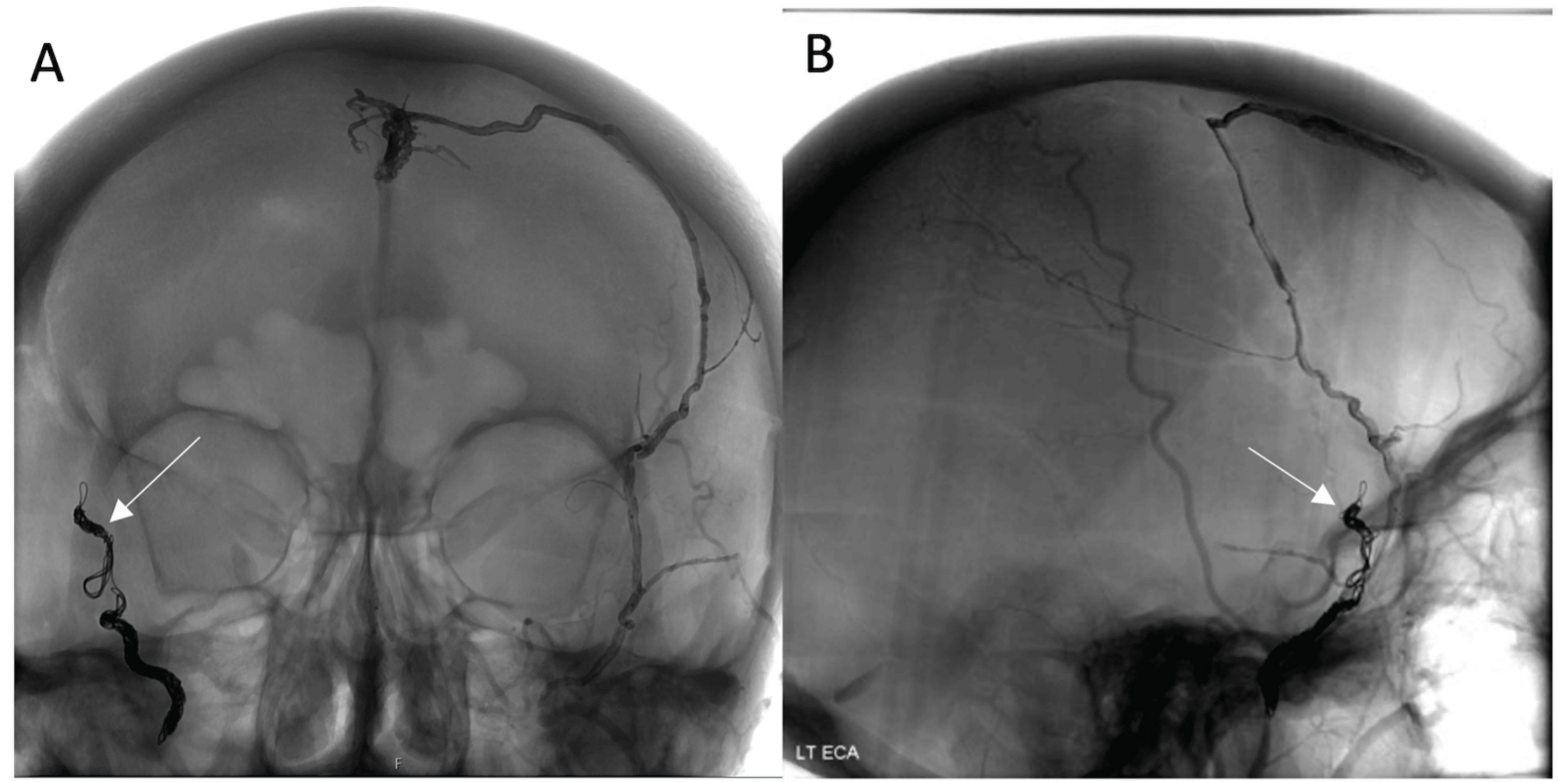
Post-coiling digital subtraction angiogram (DSA). AP (A) and lateral (B) digital subtraction angiogram views demonstrating embolization of the left MMA. Right MMA occluded with coils due to intra-procedural rupture. LT ECA: left external carotid artery, AP: anterior posterior, MMA: middle meningeal artery.

This was a high-grade Cognard type IIa+b dAVF, with an 8% hemorrhage risk per annum [6]. After a second MDT discussion, an experienced interventional neuroradiologist attempted endovascular embolization. She underwent embolization of the left MMA, with attempted embolization of the right MMA, which ruptured and was occluded with coils (Figure 4).

**Figure 5:**
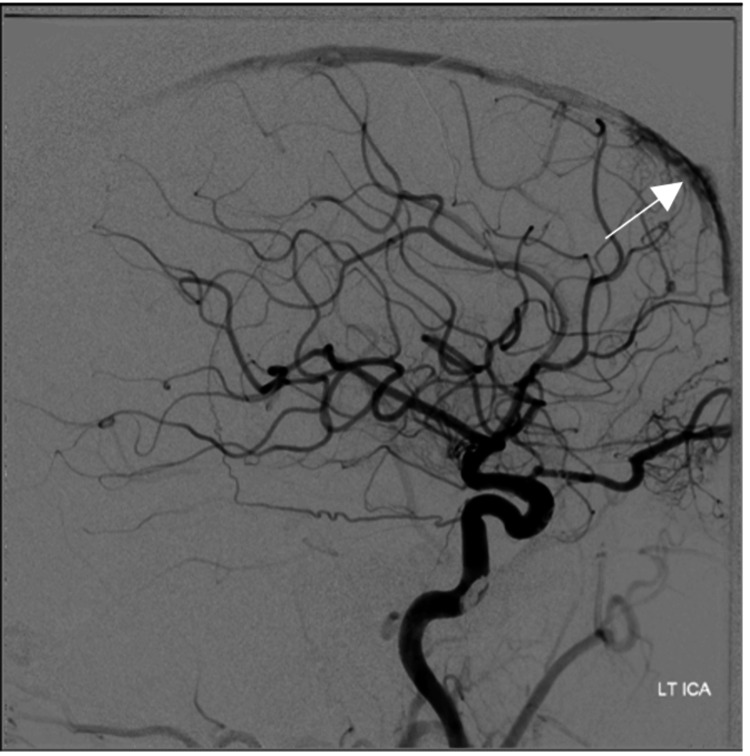
Post-embolization digital subtraction angiogram of left ICA. Demonstrates patent fistula with supply from the left anterior falcine artery. LT ICA: left internal carotid artery.

The team was unable to access and divide the left anterior falcine supply (distal left ophthalmic supply), which remained as displayed (Figure 5).

Five days later, after a further MDT discussion, she underwent a midline craniotomy and ligation of the dAVF and anterior sagittal sinus with no immediate complications. With the aid of neuronavigation (Stealth S8), a craniotomy was centered over the anterior superior sagittal sinus, with burr holes on the midline. The dura was opened on both sides of the sagittal sinus. The feeding arteries were identified, with the help of indocyanine green (ICG), and ligated, followed by division of the anterior third of the SSS. The falx was retracted and divided posteriorly, with the embolized vein marking the posterior boundary. Further division of feeders was done, including the right ophthalmic artery. The right olfactory nerve was identified and preserved. Finally, distended veins on the falx were coagulated, and meticulous hemostasis was secured.

**Figure 6:**
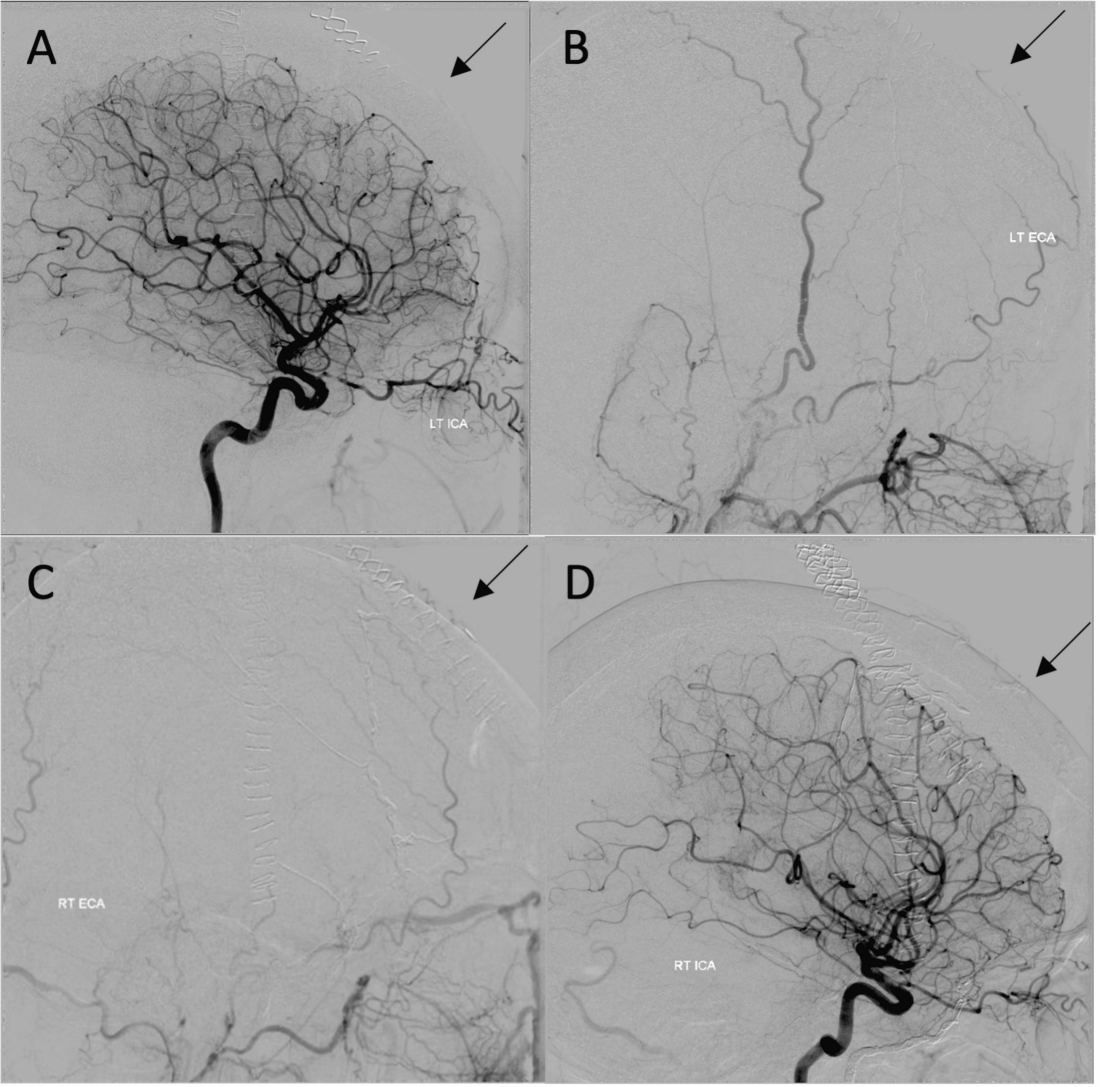
Post-operative angiogram showing occlusion of the fistula. (A) and (B): Left ICA and ESA DSA. (C) and (D): Right ESA and ICA DSA. LT ICA: left internal carotid artery, LT ECA: left external carotid artery, RT ECA: right external carotid artery, RT ICA: right internal carotid artery.

Post-operative DSA showed complete occlusion of the fistula (Figure 6). She had a loss of smell and reduced taste post-operatively, likely due to neuropraxia of the olfactory nerve. Her memory deficits transiently worsened, with improvement over time. This is likely due to the hemodynamic affects from two general anesthetics and interventions on an already hemodynamically grossly abnormal brain. She, therefore, needed help with her daily living tasks, initially with carers three times a day. 

In hindsight, with a closer examination of previous imaging, it was noted that on an MRI from 2018, she had left-sided transverse-sigmoid sinus thrombosis with a patent right-sided system and no evidence of venous hypertension. By 2020, both transverse-sigmoid sinuses were occluded. It is likely that this occlusion triggered angiogenesis and fistula formation.

She was referred to a neurologist for her extensive thrombosis, who advised restarting her clopidogrel. They also speculated that her thrombosis was related to her coeliac disease. The patient had a history of TIA, which may also suggest a hypercoagulable state. Her blood tests in 2021 showed raised serum vitamin B12 and normal folate levels, making hyperhomocysteinemia less likely. She had negative antiphospholipid antibodies and lupus anticoagulant. She had no family history of venous thromboembolism, was a non-smoker, and adhered to a gluten-free diet. However, her significant, progressive, bilateral cerebral venous sinus thrombosis while on clopidogrel suggests an underlying cause, with coeliac disease being her only risk factor.

## Discussion

We report a case of a convexity dAVF in a rare anterior SSS location. This was a Cognard IIa+b dAVF, which is a high risk, with an 8.1% and 6.9% risk of hemorrhage and non-hemorrhagic neurological deficit per annum, respectively [6]. A 2022 literature review demonstrates that SSS locations of dAVF are rare in themselves, with no cases of anterior SSS identified on their extensive review [7]. 

Due to the hemorrhage risk, the MDT prioritized urgent treatment. Endovascular treatment, aiming for a cure, proved to be a challenge. This was due to the need for a bilateral arterial approach and the anterior falcine supply, which was hard to access directly. This would have to be occluded during liquid embolic embolization, e.g., via MMA or the ophthalmic artery. SSS dAVFs are increasingly being treated via a venous approach (endovascular) [1]. However, surgery still has a prominent role. The exact site of fistulation and extent of the frontal paranasal sinus will also affect operability. In this case, endovascular treatment was attempted due to the experience of the operator, but surgery could have also been an option for the first line. 

Headache and tinnitus are common complaints to the primary care physician, and a fistula must be included in the differential [1]. Our patient had a delay of six months from the first presenting and cranial imaging. With benign dAVFs, computed tomography (CT) and MRI imaging may appear unremarkable [8]. With more aggressive fistulas, such as ours, imaging may recognize pathological sequelae associated with these lesions, for example, venous hypertension [4]. We speculate that the fistula was related to the patient’s underlying extensive, bilateral thrombosis of the transverse and sigmoid sinuses. With such gross venous congestion, it is likely that our patient had symptoms of raised intracranial pressure from the thrombosis alone before a fistula developed. This could have been diagnosed earlier, avoiding two invasive treatments and improving her quality of life.

Coeliac disease and venous thrombosis

Venous sinus thrombosis can lead to angiogenesis in an attempt at recanalization and to improve venous drainage [9]. It is theorized that the presence of a thrombus within the cerebral venous system causes blood flow stasis, hence leading to increased venous pressure [9]. This increased venous pressure leads to the enlargement of pre-existing arteriovenous shunts, which are physiological in nature, or angiogenesis, which can lead to dAVF formation, as seen in this case [9]. 

Our patient’s risk factor for thrombosis was her coeliac disease. This is a chronic inflammatory disease of the small intestine triggered by ingestion of dietary gluten [10]. Most individuals have a genetic susceptibility, commonly HLA-DQ2 or HLA-DQ8 [10,11]. Classic gastrointestinal symptoms of coeliac disease include lethargy, diarrhea, abdominal distension, and digestion impairment [11]. This leads to a variety of physiological abnormalities, including hyperhomocysteinemia, low levels of vitamin K-dependant anticoagulant factors, and increased levels of thrombin-activatable fibrinolysis inhibitor [12]. The result of this is a prothrombotic state [12]. Thrombi are most likely in the hepatic vein, with other locations, such as cerebral blood vessels, decreasing in likelihood, with a prevalence of 31% and 18%, respectively [13]. 

Literature review

This is the first published case of a dAVF secondary to thrombosis in a patient with coeliac disease. To date, only six cases (in English, in five papers) of intracranial venous sinus thrombosis associated with coeliac disease have been reported on PubMed (Table 1). 

**Table 1:** A summary of the six previously published cases of intracranial venous sinus thrombosis associated with coeliac disease, along with our case. *Presumed diagnosis based on blood results, but no biopsy obtained as the patient was <5 years old. F: female; M: male; L: left; R: right; SSS: superior sagittal sinus; TS: transverse sinus; SS: sigmoid sinus; ISS: inferior sagittal sinus; d: days; m: months; y: years; UH: unfractionated heparin; LMWH: low molecular weight heparin; GOSE: Glasgow outcome scale extended.

Author, year	Age and sex	Location of thrombosis	Presenting symptoms	Cause of thrombosis	Treatment	Follow-up	Outcome from treatment	GOSE
Singla et al., 2022 [14]	24 F	Cerebral veins, vein of Galen and straight sinus	Fever, headache, vomiting and altered sensorium	Protein S deficiency	UH 2w, warfarin	14d	No complications. No recurrence.	8
Wiem et al., 2021 [15]	44 F	SSS	Hemiplegia and status epilepticus	Protein C deficiency	LMWH 2d, warfarin 6m	3y	No complications. No recurrence.	8
Alhosain and Kouba, 2020 [16]	40 F	L TS + SS	Headache, R hemiplegia and altered mental status	Hyperhomocysteinemia	LMWH, aspirin	20d	Expressive aphasia. No recurrence.	6
Bouziane et al., 2020 [17]	17 F	R TS +SS	Headache and muscle weakness	No stated cause	LMWH then warfarin	2y	No complications. No recurrence.	8
Dogan et al., 2011 [18]	16 M	SSS, ISS, L TS + LSS	Headache and vomiting	Coeliac disease	LMWH	2m	No complications. No recurrence.	8
Dogan et al., 2011 [18]	2 M	R TS	Irritability and vomiting	Coeliac disease*	LMWH	6m	No complications.	8
Chand and Parwez, 2024	67 F	Anterior SSS	Headache, neck stiffness, L ear tinnitus of six months	Suspected coeliac disease	Clopidogrel: endovascular and surgical for fistula	2y	Complete occlusion of fistula. No complications	7

There are several limitations to this study. It is a case report of one patient only. We speculate that her coeliac disease made her prothrombotic, which triggered the progressive venous sinus thrombosis, and the development of a fistula. We have no way to prove this definitively, although it is suggested by the time course of the images. In some cases, the fistula can occur first, triggering thrombosis [19]. In western populations, coeliac disease prevalence has been estimated to be up to one in 99 children, so one may expect the relationship with thrombosis. Thus, dAVF is to be reported more highly in the literature [20]. Given the rarity of both the location and complexity of the dAVF and the relationship with coeliac disease, this is a valuable contribution to the literature. 

## Conclusions

This is an anterior superior sagittal sinus dAVF with a predominant arterial supply from the right ECA via the MMA and a lesser supply from the other arteries. The fistula drains into the rostral third of the SSS, with deep venous reflux into the right internal cerebral vein. Fistulas on the anterior SSS are rare and challenging to treat from an endovascular approach as they often have bilateral supply from both ICA and ECA sources. It is important to discuss these cases in an MDT and consider surgery as an appropriate first-line treatment.

This is the first reported case of a fistula secondary to venous sinus thrombosis in a patient with coeliac disease. It is important to be aware of the prothrombotic state of patients with coeliac and the risk of thrombosis and fistula formation. This allows physicians to recognize the signs of raised intracranial pressure, facilitating early intervention in the case of thrombosis or fistula.
